# Supplementary material for: HIVprotI: an integrated web based platform for prediction and design of HIV proteins inhibitors
Source: J Cheminform. 2018 Mar 9;10:12. doi: 10.1186/s13321-018-0266-y (PMC5845081; doi:10.1186/s13321-018-0266-y)
Supplement: Supplementary file 2 — Additional file 2. Source code of HIVProtI web server. [file 13321_2018_266_MOESM2_ESM.zip › HIVprotI_Source-code/hivprotI/weka-3-6-6/doc/weka/core/TechnicalInformationHandlerJavadoc.html]

TechnicalInformationHandlerJavadoc


---


|  |  |  |
| --- | --- | --- |
| **Overview** \| **Package** \| **Class** \| **Tree** \| **Deprecated** \| **Index** \| **Help** \| **Weka's home** | |  |
| **PREV CLASS**   **NEXT CLASS** | **FRAMES**    **NO FRAMES**     **All Classes** |  |
| SUMMARY: NESTED \| FIELD \| CONSTR \| METHOD | DETAIL: FIELD \| CONSTR \| METHOD |  |


---


## weka.core Class TechnicalInformationHandlerJavadoc

```
java.lang.Object
  weka.core.Javadoc
      weka.core.TechnicalInformationHandlerJavadoc
```

**All Implemented Interfaces:**: OptionHandler, RevisionHandler

---

``` public class TechnicalInformationHandlerJavadoc extends Javadoc ```

Generates Javadoc comments from the TechnicalInformationHandler's data.
Update the BibTex references and the plaintext techincal information.


Valid options are:

```
 -W <classname>
  The class to load.
```

```
 -nostars
  Suppresses the '*' in the Javadoc.
```

```
 -dir <dir>
  The directory above the package hierarchy of the class.
```

```
 -silent
  Suppresses printing in the console.
```

```
 -noprolog
  Suppresses the 'BibTex:' prolog in the Javadoc.
```

**Version:**
:   $Revision: 1.6 $

**Author:**
:   fracpete (fracpete at waikato dot ac dot nz)

**See Also:**: `PLAINTEXT_STARTTAG`, `PLAINTEXT_ENDTAG`, `BIBTEX_STARTTAG`, `BIBTEX_ENDTAG`

---

| **Field Summary** | |
| --- | --- |
| `static java.lang.String` | `BIBTEX_ENDTAG`             the end comment tag for inserting the generated BibTex |
| `static java.lang.String` | `BIBTEX_STARTTAG`             the start comment tag for inserting the generated BibTex |
| `static java.lang.String` | `PLAINTEXT_ENDTAG`             the end comment tag for inserting the generated BibTex |
| `static java.lang.String` | `PLAINTEXT_STARTTAG`             the start comment tag for inserting the generated BibTex |


**Constructor Summary**

`TechnicalInformationHandlerJavadoc()`
  
          default constructor


| **Method Summary** | |
| --- | --- |
| `java.lang.String[]` | `getOptions()`             Gets the current settings of this object. |
| `boolean` | `getProlog()`             whether "Valid options are..." prolog is included in the Javadoc |
| `java.lang.String` | `getRevision()`             Returns the revision string. |
| `java.util.Enumeration` | `listOptions()`             Returns an enumeration describing the available options. |
| `static void` | `main(java.lang.String[] args)`             Parses the given commandline parameters and generates the Javadoc. |
| `void` | `setOptions(java.lang.String[] options)`             Parses a given list of options. |
| `void` | `setProlog(boolean value)`             sets whether to add the "Valid options are..." prolog |

**Methods inherited from class weka.core.Javadoc**

`generate, generateHelp, getClassname, getDir, getSilent, getUseStars, setClassname, setDir, setSilent, setUseStars, updateJavadoc`

**Methods inherited from class java.lang.Object**

`equals, getClass, hashCode, notify, notifyAll, toString, wait, wait, wait`

**Field Detail**

### PLAINTEXT\_STARTTAG

```
public static final java.lang.String PLAINTEXT_STARTTAG
```

:   the start comment tag for inserting the generated BibTex

    **See Also:**: Constant Field Values

---


### PLAINTEXT\_ENDTAG

```
public static final java.lang.String PLAINTEXT_ENDTAG
```

:   the end comment tag for inserting the generated BibTex

    **See Also:**: Constant Field Values

---


### BIBTEX\_STARTTAG

```
public static final java.lang.String BIBTEX_STARTTAG
```

:   the start comment tag for inserting the generated BibTex

    **See Also:**: Constant Field Values

---


### BIBTEX\_ENDTAG

```
public static final java.lang.String BIBTEX_ENDTAG
```

:   the end comment tag for inserting the generated BibTex

    **See Also:**: Constant Field Values


**Constructor Detail**

### TechnicalInformationHandlerJavadoc

```
public TechnicalInformationHandlerJavadoc()
```

:   default constructor


**Method Detail**

### listOptions

```
public java.util.Enumeration listOptions()
```

:   Returns an enumeration describing the available options.

    :   **Specified by:**: `listOptions` in interface `OptionHandler` **Overrides:**: `listOptions` in class `Javadoc`
    :   **Returns:**: an enumeration of all the available options.

---


### setOptions

```
public void setOptions(java.lang.String[] options)
                throws java.lang.Exception
```

:   Parses a given list of options.

    :   **Specified by:**: `setOptions` in interface `OptionHandler` **Overrides:**: `setOptions` in class `Javadoc`
    :   **Parameters:**: `options` - the list of options as an array of strings **Throws:**: `java.lang.Exception` - if an option is not supported

---


### getOptions

```
public java.lang.String[] getOptions()
```

:   Gets the current settings of this object.

    :   **Specified by:**: `getOptions` in interface `OptionHandler` **Overrides:**: `getOptions` in class `Javadoc`
    :   **Returns:**: an array of strings suitable for passing to setOptions

---


### setProlog

```
public void setProlog(boolean value)
```

:   sets whether to add the "Valid options are..." prolog

    :   **Parameters:**: `value` - true if the prolog is to be used

---


### getProlog

```
public boolean getProlog()
```

:   whether "Valid options are..." prolog is included in the Javadoc

    :   **Returns:**: whether the prolog is currently used

---


### getRevision

```
public java.lang.String getRevision()
```

:   Returns the revision string.

    :   **Returns:**: the revision

---


### main

```
public static void main(java.lang.String[] args)
```

:   Parses the given commandline parameters and generates the Javadoc.

    :   **Parameters:**: `args` - the commandline parameters for the object


---


|  |  |  |
| --- | --- | --- |
| **Overview** \| **Package** \| **Class** \| **Tree** \| **Deprecated** \| **Index** \| **Help** \| **Weka's home** | |  |
| **PREV CLASS**   **NEXT CLASS** | **FRAMES**    **NO FRAMES**     **All Classes** |  |
| SUMMARY: NESTED \| FIELD \| CONSTR \| METHOD | DETAIL: FIELD \| CONSTR \| METHOD |  |


---
